# Efficacy of *Rubus coreanus* Miq. and *Astragalus membranaceus* Bunge Extract for Postmenopausal Syndrome: A Randomised, Double-Blind, Placebo Comparative Clinical Trial

**DOI:** 10.1155/2022/4066054

**Published:** 2022-02-23

**Authors:** Jeong-Su Park, Hyun Kyung Sung, Seul-Ki Kim, Hak Sung Lee, Seon Mi Shin

**Affiliations:** ^1^Department of Preventive Medicine, College of Korean Medicine, Semyung University, Semyeong-ro 65, Jecheon-si, Chungcheongbuk-do 27136, Republic of Korea; ^2^Department of Pediatrics, College of Korean Medicine, Semyung University, Semyeong-ro 65, Jecheon-si, Chungcheongbuk-do 27136, Republic of Korea; ^3^Skin & Natural Products Lab, Kolmar BNH, Heolleung-ro 8-gil, Seocho-gu, Seoul, Republic of Korea; ^4^Department of Korean Internal Medicine, College of Korean Medicine, Semyung University, Semyeong-ro 65, Jecheon-si, Chungcheongbuk-do 27136, Republic of Korea

## Abstract

**Objectives:**

The most effective way to improve menopausal symptoms is to supplement deficient oestrogen; however, long-term administration of synthetic oestrogen increases the risk for breast and uterine cancers. Here, we report results from a clinical trial of *Rubus coreanus* Miq. and *Astragalus membranaceus* Bunge as agents for improving the menopause syndrome.

**Methods:**

This study was a single-centre, double-blinded, parallel-group, placebo-controlled study. The efficacy of an extract of *R. coreanus* Miq. and *A. membranaceus* Bunge was investigated. Participants were females with postmenopausal syndrome in the menopausal or menopausal transition period. The primary endpoint of validation was improvement in the Kupperman index (KI) score of women. The secondary end point was change in the Menopause Rating Scale (MRS) and lipid profile. The participants were randomly allocated at a 1 : 1 ratio into *R. coreanus* Miq. and *A. membranaceus* Bunge extract (RCAM) or placebo groups and were administered 2000 mg of the extract or placebo, respectively, daily for 12 weeks. Outcomes were measured at visits 2 (day 0) and 5 (week 12).

**Results:**

The RCAM group demonstrated decreased KI score and MRS compared with the placebo group after 12 weeks. In the safety evaluation, laboratory tests and vital signs demonstrated no clinically significant changes in subjects, and there was no difference in adverse reactions between the groups. The *R. coreanus* Miq. and *A.* membranaceus Bunge extract was effective in reducing postmenopausal symptoms in women. Moreover, the extract was found to be safe.

**Conclusions:**

For females with menopausal symptoms in the menopausal transitional and postmenopausal periods, ingestion of the *R. coreanus* Miq. and *A. membranaceus* Bunge extract for 12 weeks was effective, as demonstrated by a decrease in KI score and MRS relative to that in the placebo group, and significantly improved the menopausal symptoms.

## 1. Introduction

After menopause, because of the rapid decline in oestrogen, women are prone to a significantly increased risk of developing psychological and emotional symptoms such as fatigue, excitation, insomnia, poor concentration, depression, memory loss, headache, anxiety, and sleep disturbances. Physical symptoms can include recurrent facial flushing, intermittent dizziness, sensory abnormalities, heart disease, and nausea. Osteoporosis also becomes more common, and mortality from cardiovascular diseases, including heart disease, high blood pressure, and stroke, increases [1]. Menopause also induces changes in the skin and female urogenital system, and the likelihood of developing autoimmune diseases, cataracts, and colon cancer increases. Currently, the most effective way to improve the above menopausal symptoms is to supplement the deficient oestrogen. However, long-term administration of synthetic oestrogen increases the risk of breast and uterine cancer [2–4]. There is a growing controversy among scholars and throughout society that long-term synthetic oestrogen replacement therapy causes breast cancer. Although not robustly demonstrated, the breast cancer risk in women undergoing synthetic oestrogen replacement therapy is believed to be related to oestrogen exposure over time. Therefore, finding a phytoestrogen, a vegetable-based oestrogen, as a safer substitute for synthetic oestrogen for hormone replacement therapy has become an active area of research. Phytoestrogens are nonsteroidal compounds present in plants that have oestrogenic effects in animal bodies. Most of the grains, fruits, and vegetables known to have anticancer and anti-heart-disease properties contain trace amounts of phytoestrogens. There has been a steady need for safe drugs or health-promoting foods that are also effective in improving menopause symptoms in women and do not increase the risk of breast or uterine cancer. In this context, we conducted a clinical trial of an extract containing *Rubus coreanus* Miquel and *Astragalus membranaceus* Bunge to evaluate its ability to improve the postmenopausal syndrome.

## 2. Methods and Design

### 2.1. Study Participants

**Figure 1:**
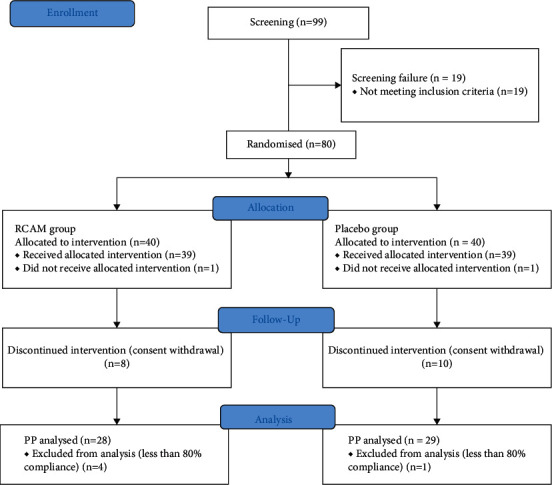
The study flow chart. The intervention: the *Rubus coreanus* Miq. and *Astragalus membranaceus* Bunge extract and placebo (RCAM: *Rubus coreanus* Miq. and *Astragalus membranaceus* Bunge).

Healthy subjects were recruited through a written notice posted on the hospital homepage and bulletin board of Semyung University Korean Medicine Hospital (Jecheon, Chungcheongbuk-do, the Republic of Korea) until the target sample size was reached. Women willing to participate in the study voluntarily visited the Department of Internal Medicine and were enrolled, after providing informed consent, if they met the inclusion criteria. The first participant was enrolled in September 2019. This clinical trial protocol (ver. 1.4; issue date, 2019) was approved by the Institutional Review Board of Semyung University Korean Medicine Hospital (approval no. SMJOH-2019-09-01) and registered at the Korean Clinical Research Information Service (KCT0005546).We followed the methods of Song et al. 2019 [5]. The participants fulfilled all the criteria mentioned in suppl.  Table 1. The total time for study participation was approximately 16 weeks, which included consumption of the investigational product for 12 weeks, a maximum of a 2-week washout period, and safety assessment 2 weeks after the last visit. Written informed consent was provided, and inclusion and exclusion criteria were assessed at the first visit. If a participant had a drug history of concomitant use of prohibited medication or food, a maximum 14-day washout period was required, for which the participant also had to provide consent. Participants were randomised into the treatment or control group during visit 2, which served as the baseline time point. The inclusion and exclusion criteria were rechecked before randomisation, and then participants who met the criteria were enrolled. A baseline assessment was performed and 33-day supplies of investigational products or placebos were provided to the participants. Follow-up visits occurred 28 (visit 3), 56 (visit 4), and 84 (visit 5) days after the baseline assessment (visit 2). A five-day visit window was allowed. Checking of vital signs, drug history/concomitant drug examination, laboratory tests, pregnancy test, efficacy and safety evaluation, and additional provision of investigational products were performed during visits 3–5 (Figure 1 and suppl.  Table 2). The schedule was notified to the hospital visitors through an announcement.

### 2.2. Interventions

**Figure 2:**
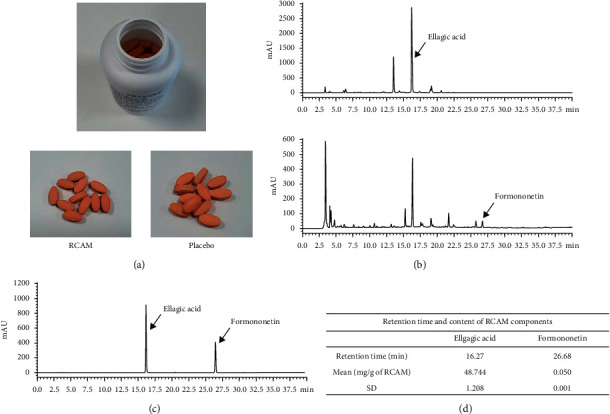
(a) The RCAM tablet and placebo. (b, c, d) Fingerprinting analysis of RCAM. (b) Chromatogram of ellagic acid and formononetin. (c) Chromatogram of standard mixture. (d) The content of each standard compound in RCAM.

The investigation was conducted by Kolma Inc. (Jecheon, Chungcheongbuk-do, the Republic of Korea). A 1,000 mg tablet contained powder of 48% *R. coreanus* Miq. and *A. membranaceus* BUNGE extract mixed at a ratio of 7 : 3 (w/w), 2 tablets (2,000mg) per day. The place of origin of *R. coreanus* Miq. was Gwangyang, and that of *A. membranaceus* Bunge was Jecheon (the Republic of Korea). The fruit of *R. coreanus* Miq. and root of *A. membranaceus* Bunge were extracted with 30% ethanol for 4 hours and then at 70°C for 2 hours. The extract was resistant to any change in the gastric environment. The extract was evaporated, lyophilised, and then stored at −75°C. The final product was made into tablet form (Figure 2). The intervention and placebo tablets were identical in shape, size, and colour. Participants were orally administered either two intervention or two placebo tablets per dose, twice a day (morning and evening after meals) for 12 weeks. They received a one-month supply of intervention at baseline and during weeks 4 and 8 and were encouraged to continue following the prescribed dosage regimen. At weeks 4, 8, and 12, the unused tablets were returned and counted to evaluate participants' compliance. Participants were advised to maintain their usual diet and exercise levels during the study. They were prohibited from taking medicines or consuming foods that could affect their body weight and body fat during the study. Medicines, foods, exercise therapy, and diets that participants maintained prior to the enrolment were allowed at the investigator's discretion. Drugs and foods that could have directly or indirectly affected the results of this study (factors, such as drugs, food, and health functional foods, which may affect menopausal symptoms) were not to be consumed during the study period. The contraindicated drugs and foods were as follows: (1) contraindicated drugs: sleep inducers, antidepressants, selective oestrogen receptor modulators, antihyperlipidemic agents, and antihypertensives; (2) contraindicated functional foods: functional foods that help improve the health of menopausal women (such as soybean isoflavones, *Linum usitatissimum*, *Punica granatum* L., *Cimicifuga heracleifolia* Kom., *Cynanchum wilfordii* H., and *Trifolium pratense*). During the study period, if a subject wanted to consume drugs or food due to specific circumstances, they needed prior consultation with the researcher. Information regarding all concomitant medications, including the product or ingredient name, dosage, and duration, was recorded at every visit. Intervention was discontinued if (1) a serious adverse event occurred; (2) a participant used a drug or underwent a physical procedure that could affect menopausal symptoms; (3) a participant wished to discontinue participation; (4) difficulties in assessment occurred for administrative reasons, such as violations of dosage method or visit schedule; (5) difficulties in follow-up occurred owing to a participant's personal reasons.

### 2.3. Randomization and Blinding

Participants determined to be eligible for this study were randomly assigned to either the *R. coreanus* Miq. and *A. membranaceus* Bunge extract (RCAM) groups or the placebo group at a 1 : 1 ratio using a computerised block randomisation method. The randomisation method used was stratified permuted block randomisation, and the randomisation table was a table in which the statistician applied the permutation of random numbers generated in the *R* project for statistical computing sequentially from subject number 1. A randomisation table was prepared separately so that the ratio between the test group and the control group could be assigned 1 : 1 (e.g., no. 1 = *A* = RCAM group; no. 2 = *B* = placebo group). Block sizes ranged from 2 to 8. The randomisation table was prepared with twice the number of planned subjects in each group. An independent statistician generated the randomisation sequence numbers, and the block size was not disclosed to the investigator for allocation concealment. Participants were consecutively assigned a randomisation number according to the enrolment order and received their investigational product labelled with the same randomisation number throughout the trial. The packaging and labels were identical for the two groups for blinding purposes. Participants and all research personnel were blinded to the group assignment. The randomisation sequence was concealed in sealed opaque envelopes and was not to be disclosed to the investigator until the end of the study, except when crucial, such as if a serious adverse event occurred. If a code-break occurred, the investigator had to notify the contract research organisation and the sponsor immediately, and the date and reason for the code-break were recorded in the case-report form. The mechanism of implementing the allocation sequence involved sequential numbering and the use of opaque and sealed envelopes. Mediation practitioners generated the allocation sequence, enrolled participants, and assigned participants to the interventions. Trial participants, outcome assessors, and data analysts were blinded after assignment to the interventions; if an adverse reaction occurred, the intervention operator was to disclose the intervention to the principal investigator.

### 2.4. Outcome Measures and Endpoints

The primary endpoint was the mean difference in the Kupperman Index (KI) score between visit 2 (day 0) and visit 5 (week 12) in menopausal and menopausal transition females. The KI score was a questionnaire tool that assessed 11 symptoms and characteristics of the menopausal period, including hot flashes, insomnia, nervousness, paraesthesia, arthralgia, fatigue, headache, heart palpitation, melancholia, vertigo, and formication. Each symptom was scored on a four-point scale of 0 (no symptoms) to 3 (very painful). The weighting factor was 4 for hot flashes and 2 for nervousness, paraesthesia, and insomnia. The KI scores range from 0 to 51.

The secondary endpoints were efficacy and safety. The efficacy endpoint included the change in the Menopause Rating Scale (MRS) and fasting lipid profile (total cholesterol (TC), triglycerides (TG), low-density lipoprotein cholesterol (LDL-C), and high-density lipoprotein cholesterol (HDL-C)) between visit 2 (day 0) and visit 5 (week 12). The MRS was used to estimate the quality of life during the menopausal period. It consisted of psychological, physical, and urogenital symptoms using three domains and 11 items. The safety endpoint covered intake compliance, adverse events (such as frequency, characteristics, and severity), differences in endometrial thickness, and levels of estradiol, follicle stimulating hormone, and luteinizing hormone.

### 2.5. Sample Size

A sample size calculation was performed assuming a change in KI scores between baseline and week 12 with a mean difference of 11.0 and a standard deviation of 15.7 [6]. Anticipating a 20% drop-out rate, a total sample size of 80, with 40 participants per group, was required.

### 2.6. Statistical Analyses

The independent two-sample, two-tailed *t*-test was used to compare the changes between the baseline and week 12. A *p*value ≤0.05 was considered statistically significant. Analyses were performed using intention-to-treat (ITT) and per-protocol (PP) datasets.

For all outcomes observed in this study, the improvement compared to the baseline value was calculated and descriptive statistics (such as mean, standard deviation, median, minimum, and maximum) are presented. After checking for the normal distribution of data, the independent *t*-test or Wilcoxon's rank test for the intervention and placebo groups were analysed using the rank-sum test. Adverse reactions were evaluated by calculating the incidence of all adverse reactions reported during the study period. The proportion of subjects who had an adverse reaction in each group was calculated and compared and analysed using the chi-square or Fisher's exact test.

## 3. Results

This clinical trial was conducted from the date of the first subject's visit on 22 October 2019 to the last subject's completion date on 22 August 2020. A total of 99 subjects were enrolled for this clinical trial, and a total of 57 subjects completed the trial; 28 subjects in the RCAM group and 29 subjects in the placebo group (Figure 1 and Table 1).

### 3.1. Primary Outcome

The KI score, which was the primary outcome, was significantly decreased in the RCAM group compared with that in the placebo group (Table 2).

### 3.2. Secondary Outcomes

There was a significant difference between the groups in the amount of change in the MRS, which was a secondary outcome (*p* = 0.005). There was no statistically significant difference between the groups in the lipid index (TG, LDL-C, and HDL-C), except for that in TC (Table 2).

### 3.3. Safety

There were no clinically significant changes between the groups in the vital signs, blood tests, and urine tests except for AST and ALT. A comparison of the 12-week and baseline values for the RCAM and placebo groups showed a significant difference in AST and ALT levels at visit 5, but the values were within the normal range (0–35 IU/L) (Table 2).

There were 14 adverse reactions in 13 subjects (16.66%) who experienced more than one adverse reaction during the clinical trial period. There was no statistically significant difference between the groups in the occurrence of adverse reactions. There were no clinical symptoms of any serious adverse reaction. With regard to safety assessment, we assessed the effects of the investigation on the endocrine function and thickness of the endometrium through ultrasound examinations at the baseline and visit 5, and measured the levels of estradiol, follicle stimulating hormone (FSH), and luteinizing hormone (LH); no statistically significant change in these parameters was observed between the baseline (visit (2) and visit 5) (Table 2).

## 4. Discussion

Menopause refers to all stages of menopause and the postmenopausal period, during which female hormones change, especially when oestrogen levels decrease, leading to various menopause symptoms that can vary in type, severity, and duration. The most common symptoms that cause women to seek medical care include urogenital symptoms, vaginal dryness, vascular system-related symptoms, and hot flashes. Furthermore, not only do mental and psychological symptoms, such as sleep disorders, depression, and anxiety, become more frequent, but also the risk of chronic diseases, such as cardiovascular disease and osteoporosis, increases during menopause.

*R. coreanus*, one of the materials used in the present research, produces an immature fruit called bokbunja (覆盆子). In Korea, *R. coreanus* Miq. is dried in the sun and can then be mixed with drugs, alcohol, or juice. It has been used as a traditional Korean medicinal ingredient for strengthening liver function, kidney function, and eyesight [7] and is known to clear the eyes, provide diuretic efficacy, and treat stamina depletion and frequent urination. It has also been reported to have physiological benefits, including antioxidant effects, antifatigue effects, prevention of osteoporosis and diabetes, and female hormone replacement [8, 9]. Recently, Kim et al. [10] reported a balanced recovery of blood lipids and arteriosclerosis prevention effects of an extract of immature *R. coreanus* Miq fruit administered to mice fed a high-fat diet for 14 weeks. According to a study by Ju et al. [11], the phenolic acids in *R. coreanus* Miq extract include gallic acid, protocatechuic acid, p-hydroxybenzoic acid, vanillic acid, syringic acid, p-coumaric acid, ferulic acid, salicylic acid, and rosmarinic acid. Extracts of *R. coreanus* in most solvents, including aqueous acetone and ethyl acetate, have the highest protocatechuic acid content. *R. coreanus* Miq showed dual regulation of osteoblasts and osteoclasts to maintain the bone turnover rate in postmenopausal osteoporosis [12].

*Astragali* Radix, the other material used in this study, is the root of the perennial leguminous herb, *A. membranaceus* Bunge. The peeled and dried root is an important medicinal ingredient that is used in about 300 kinds of Chinese medicines, including Hwanggigunjungtang (黃芪建中湯), Banggihwanggitang (防己黃芪湯), and Bojungikgitang (補中益氣湯). The main components of *A. membranaceus* Bunge are isoflavonoids and triterpene saponin; it also contains phenol glycoside and *γ*-aminobutyric acid. The health-promoting properties of *A. membranaceus* Bunge have been evaluated in several studies; these include diuretic effects, the ability to decrease blood pressure, improve cellular and humoural immunity, anti-inflammatory effects, and the ability to reduce alkaline RNase activity in the liver and spleen. A few side effects of *A. membranaceus* Bunge have been reported, and it is often added to food as a supplement [13]. *A. membranaceus* Bunge is known for several effects, such as anti-inflammatory, antifatigue, antidiabetic, and antiosteoporosis effects [14]. The *R. coreanus* Miq. and *A. membranaceus* Bunge extract demonstrated oestrogen activity and promoted the growth of osteoblasts in an experimental study. It also improved menopausal symptoms in a mouse model of ovariectomy and improved the symptoms in females without causing adverse reactions [15]. In a 12-week placebo-controlled study on symptom improvement in subjects with menopausal symptoms, KI score and MRS were improved in subjects in the RCAM group compared with those in the placebo group. In the safety evaluation, there were no clinically significant changes in the results of general blood and urine tests. There was a statistically significant difference between the groups in albumin levels at 12weeks compared with the baseline levels, but the level was within the normal range. The original KI has been used for several decades to assist physicians in summarising the severity of climacteric complaints [16]. However, it has been criticised for including some non-specific items, such as headache and vertigo, which are considered to be of little practical relevance and for not including urogenital symptoms, which are now believed to be common in menopausal women [17–19]. On the contrary, the MRS uses standardised scales to measure the severity of symptoms of aging and the impact of these symptoms on health-related quality of life. In addition, improvement of related markers and clinical indicators was observed, confirming that they are safe and effective functional materials as nutraceuticals [15, 20]. Nutraceuticals are of vegetable or animal origin. Globally, research is being conducted to obtain insights into the mechanism of action of nutraceuticals and to determine their safety and efficacy by substantiating clinical data [20]. The potential of nutraceuticals to prevent and/or support pharmacological treatments, which are primarily drug-based today, will make them a powerful tool to confront pathological, chronic, and long-term diseases in subjects ineligible for pharmacological treatment [20]. This extract is expected to be a good material for improving symptoms in menopausal females. This study has several limitations. It was a clinical trial at a single centre, and the primary end point was a subjective evaluation index. However, this is the first study evaluating the efficacy of natural products, *R. coreanus* Miq. and *A. membranaceus* Bunge, in reducing menopausal symptoms, and this clinical trial provides useful information for future research on menopausal syndrome. If multicentre and crossover studies are conducted in the future, clearer and more reliable results are expected.

## 5. Conclusion

This clinical trial was aimed at evaluating the effectiveness and safety of *R. coreanus* Miq. and *A. membranaceu*s Bunge extracton menopausal symptoms in menopausal transitional and postmenopausal women. In the RCAM group, the KI Index, used as the primary end point, was significantly decreased compared with that in the placebo group (*p* = .007). Moreover, there was a statistically significant change in the MRS, the secondary endpoint, in the RCAM group compared with the placebo group (*p* = 0.05).

In conclusion, in menopausal transitional and postmenopausal women with menopausal symptoms, the ingestion of *R. coreanus* Miq. and *A. membranaceus* Bunge extract for 12 weeks improved the menopausal symptoms. Safety evaluation, laboratory tests, and vital signs revealed no clinically significant changes in the measured indicators, and there was no difference in adverse reaction-related indicators between the groups. Therefore, the *R. coreanus* Miq. and *A. membranaceus* Bunge extract is effective in improving the symptoms and quality of life in menopausal females and is safe.

## Figures and Tables

**Table 1:** Demographic characteristics of subjects (ITT set).

	RCAM group (*n* = 40)	Placebo group (*n* = 40)	^#^ *p*value
	Mean (SD)	Mean (SD)	
Age	54.3 (3.250)	55.4 (3.134)	.136
Height	156.8 (5.767)	157.5 (5.319)	.586
Weight	58.8 (9.148)	60.9 (8.713)	.279

**Table 2:** Outcomes and safety (PP set).

		RCAM group	Placebo group	^#^ *p*value
		Mean (SD)	Mean (SD)	
Primary outcome				
Kupperman index	Visit 2	29.00 (5.722)	30.24 (7.034)	.467
	Visit 5	13.82 (6.645)	20.00 (9.666)	.007^*∗∗*^

Secondary outcome				
MRS	Visit 2	20.39 (8.052)	23.79 (6.383)	.082
	Visit 5	9.36 (4.893)	13.45 (5.736)	.005^*∗∗*^
Total cholesterol	Visit 2	215.43 (36.613)	199.83 (35.783)	.467
	Visit 5	220.71 (34.115)	211.52 (42.524)	.007^*∗∗*^
TG	Visit 2	99.00 (46.555)	141.31 (82.295)	.021^*∗*^
	Visit 5	129.04 (64.766)	155.76 (85.061)	.189
LDL-C	Visit 2	120.46 (22.401)	113.75 (25.182)	.293
	Visit 5	126.00 (25.197)	122.41 (31.072)	.635
HDL-C	Visit 2	64.61 (16.176)	55.59 (12.191)	.022^*∗*^
	Visit 5	63.25 (12.539)	56.97 (11.912)	.057

Safety				
AST (GOT)	Visit 2	27.49 (16.217)	26.13 (6.808)	.637
	Visit 5	22.58 (3.519)	25.07 (4.315)	.016^*∗*^
ALT (GPT)	Visit 2	21.59 (12.156)	23.15 (10.067)	.538
	Visit 5	17.77 (5.025)	23.50 (9.591)	.006^*∗*^
*γ*GTP	Visit 2	20.67 (14.930)	22.31 (17.535)	.658
	Visit 5	17.71 (5.917)	21.80 (11.376)	.087
ALP	Visit 2	71.03 (18.876)	76.08 (20.958)	.267
	Visit 5	68.84 (16.448)	77.57 (21.188)	.077
Total protein	Visit 2	0.73 (0.270)	0.66 (0.213)	.192
	Visit 5	0.78 (0.338)	0.71 (0.221)	.347
Albumin	Visit 2	4.58 (0.195)	4.47 (0.198)	.015^*∗*^
	Visit 5	4.55 (0.196)	4.55 (0.191)	.972
BUN	Visit 2	15.58 (4.096)	14.60 (3.145)	.240
	Visit 5	16.08 (4.935)	16.08 (3.527)	.995
Creatinine	Visit 2	0.74 (0.080)	0.74 (0.098)	.678
	Visit 5	0.73 (0.077)	0.75 (0.113)	.529
Uric acid	Visit 2	4.45 (0.882)	4.35 (0.820)	.634
	Visit 5	4.35 (0.841)	4.22 (0.875)	.551
Glucose	Visit 2	96.08 (18.190)	94.36 (13.443)	.637
	Visit 5	93.32 (14.358)	93.93 (12.029)	.858
CRP	Visit 2	0.12 (0.213)	0.11 (0.157)	.795
	Visit 5	0.08 (0.080)	0.12 (0.266)	.440
ESR	Visit 2	9.77 (4.732)	10.28 (4.963)	.642
	Visit 5	9.13 (2.323)	9.23 (3.441)	.889
Endometrial thickness	Visit 2	2.43 (1.049)	2.49 (0.867)	.761
	Visit 5	2.47 (1.002)	2.35 (0.951)	.621
E2	Visit 2	13.76 (48.753)	12.10 (27.094)	.853
	Visit 5	14.20 (29.215)	13.45 (28.678)	.920
FSH	Visit 2	79.00 (23.254)	77.53 (26.863)	.796
	Visit 5	80.41 (23.182)	76.88 (21.781)	.543
LH	Visit 2	39.66 (13.913)	35.23 (15.965)	.195
	Visit 5	40.18 (13.393)	38.54 (14.341)	.647

*p* < .05 ^*∗*^, *p* < .01 ^*∗∗*^, and *p* < .001 ^*∗∗∗*^. #: independent two-sample *t*-test, the RCAM group vs. the placebo group. †: paired *t*-test, visit 2 vs. visit 5.

## Data Availability

The data used to support the findings of this study are restricted by the Institutional Review Board of Semyung University Korean Medicine Hospital in order to protect participants privacy. Data are available from Seon Mi Shin (bunggujy21@hanmail.net) and Seul-Ki Kim (lovesshot@kolmarbnh.co.kr) for researchers who meet the criteria for access to confidential data.

## Supplementary Materials

Supplementary Materials Supplement File: the inclusion and exclusion criteria, test schedule, abbreviations, and glossary of terms.

## References

[1] Santoro N., Epperson C. N., Mathews S. B. (2015). Menopausal symptoms and their management. *Endocrinology and Metabolism Clinics of North America*.

[2] Tamimi R. M., Hankinson S. E., Chen W. Y., Rosner B., Colditz G. A. (2006). Combined estrogen and testosterone use and risk of breast cancer in postmenopausal women. *Archives of Internal Medicine*.

[3] Gertig D. M., Fletcher A. S., English D. R., Macinnis R. J., Hopper J. L., Giles G. G. (2006). Hormone therapy and breast cancer: what factors modify the association?. *Menopause*.

[4] Strom B. L., Schinnar R., Weber A. L. (2006). Case-control study of postmenopausal hormone replacement therapy and endometrial cancer. *American Journal of Epidemiology*.

[5] Song J., Shin S. M., Kim H. (2019). Efficacy and safety of HT048 and HT077 for body fat and weight loss in overweight adults: a study protocol for a double-blind, randomized, placebo-controlled trial. *Medicine (Baltimore)*.

[6] Ahn K. H., Kim S. M., Yi K. W. (2010). The effect of pomegranate on postmenopausal syndrome: a randomized, double-blind, placebo-controlled trial. *Journal of the Korean Society for Microbiology*.

[7] Kim E. J., Lee Y. J., Shin H. K., Park J. H. (2005). Induction of apoptosis by the aqueous extract of Rubus coreanum in HT-29 human colon cancer cells. *Nutrition (Burbank, Los Angeles County, Calif.)*.

[8] Jung K.-A., Han D., Kwon E.-K., Lee C.-H., Kim Y.-E. (2007). Antifatigue effect of Rubus coreanus Miquel extract in mice. *Journal of Medicinal Food*.

[9] Lee K. H., Choi E. M (2006). Rubus coreanus Miq. extract promotes osteoblast differentiation and inhibits bone-resorbing mediators in MC3T3-E1 cells. *The American Journal of Chinese Medicine*.

[10] Kim S., Kim C.-K., Lee K.-S. (2013). Aqueous extract of unripe Rubus coreanus fruit attenuates atherosclerosis by improving blood lipid profile and inhibiting NF-*κ*B activation via phase II gene expression. *Journal of Ethnopharmacology*.

[11] Ju H. K., Cho E. J., Jang M. H. (2009). Characterization of increased phenolic compounds from fermented Bokbunja (Rubus coreanus Miq.) and related antioxidant activity. *Journal of Pharmaceutical and Biomedical Analysis*.

[12] Do S. H., Lee J.-W., Jeong W.-I. (2008). Bone-protecting effect of Rubus coreanus by dual regulation of osteoblasts and osteoclasts. *Menopause*.

[13] Lu S., Chen K.-j., Yang Q.-y., Sun H.-r. (2011). Progress in the research of Radix Astragali in treating chronic heart failure: effective ingredients, dose-effect relationship and adverse reaction. *Chinese Journal of Integrative Medicine*.

[14] Durazzo A., Nazhand A., Lucarini M. (2021). Astragalus (Astragalus membranaceus Bunge): botanical, geographical, and historical aspects to pharmaceutical components and beneficial role. *Rendiconti Lincei. Scienze Fisiche e Naturali*.

[15] Koo H. J., Sohn E.-H., Kang S.-C. (2013). The optimal combination of the mixture of unripe Rubus coreanus and Astragalus membranaceus in the activation and differentiation of osteoblastic cells. *Korean Journal of Polar Research*.

[16] Schneider H. P. G., Heinemann L. A. J., Rosemeier H.-P., Potthoff P., Behre H. M. (2000). The menopause rating scale (MRS): reliability of scores of menopausal complaints. *Climacteric*.

[17] Schneider H. P. G., Heinemann L. A. J., Rosemeier H.-P., Potthoff P., Behre H. M. (2000). The menopause rating scale (MRS): comparison with Kupperman index and quality-of-life scale SF-36. *Climacteric*.

[18] Alder E. (1998). The Blatt-Kupperman menopausal index: a critique. *Maturitas*.

[19] Teng Y., Tao H., Shao C., Li Y. (2013). Correlation between the modified kupperman index and the menopause rating scale in Chinese women. *Patient Preference and Adherence*.

[20] Durazzo A., Lucarini M., Santini A. (2020). Nutraceuticals in human health. *Foods*.

