# Supplementary material for: Efficacy of Rubus coreanus Miq. and Astragalus membranaceus Bunge Extract for Postmenopausal Syndrome: A Randomised, Double-Blind, Placebo Comparative Clinical Trial
Source: Evid Based Complement Alternat Med. 2022 Feb 23;2022:4066054. doi: 10.1155/2022/4066054 (PMC8890844; doi:10.1155/2022/4066054)
Supplement: Supplementary Materials — Supplement File: the inclusion and exclusion criteria, test schedule, abbreviations, and glossary of terms. [file 4066054.f1.docx]

| The inclusion criteria |
| --- |
| 1. Female, aged 45–60 years |
| 2. Females who were either menopausal (amenorrhoea for 12 consecutive months) or who were in a menopausal transition period (amenorrhoea three consecutive months and FSH levels >40 IU/L) |
| 3. Females with menopausal symptoms of moderate or severe degree (Kupperman Index score ≥20) |
| 4. Able to provide written informed consent |
| The exclusion criteria |
| 1. Clinically significant abnormality in mammography (breast imaging reporting and data system category (BI-RADS) score of 0 or above 3. Those with BI-RADS score of 0 were enrolled based on the researchers’ discretion) |
| 2. Endometrial thickness >5 mm, as assessed by vaginal ultrasonography |
| 3. History of surgery- or chemotherapy-induced amenorrhoea, undiagnosed vaginal haemorrhage |
| 4. History of hormone therapy in six months prior to the first visit |
| 5. History of continuous consumption of health-promotional food or use of herbal medicine affecting menopausal health, blood lipids (TG, cholesterol, etc.), blood pressure, or blood flow in the month prior to the first visit |
| 6. History of taking a sleep inducer, antidepressant, selective oestrogen receptor modulator, lipid-lowering drugs, or antihypertensive drugs |
| 7. History of endometrial hyperplasia, uterine endometrial cancer, or sex-steroid-dependent organ tumours |
| 8. History of severe migraine headache, thromboembolic disorders, cerebrovascular disorders, or serious cardiovascular condition within one year before the first visit |
| 9. Uncontrolled underlying disease including liver or renal function failure (ALT or AST >3-fold higher than the reference level, creatinine >2.0 mg/dL), hypertension (systolic blood pressure >160 mmHg or diastolic blood pressure >100 mmHg), thyroid disease, diabetes, or hyperlipidaemia |
| 10. Active addiction to drugs or alcohol |
| 11. Habitual use of large amounts of isoflavone |
| 12. Continuous intensive exercise within three months prior to the first visit (≥10 hours/week) |
| 13. Hypersensitivity to the investigational product or ingredients in the investigational product |
| 14. Participation in another clinical study within the month prior to the first visit |
| 15. Inappropriate for the clinical trial according to the researchers’ discretion |

**Supplementary Table 1.** The inclusion and exclusion criteria

**Supplementary Table 2.** Test schedule

| Test items | | Screening | Intake period | | | | F/U  (if necessary) |
| --- | --- | --- | --- | --- | --- | --- | --- |
|  |  | Visit 1 | Visit 2 | Visit 3 | Visit 4 | Visit 5 |  |
|  |  | -2 weeks | 0 week | 4 weeks | 8 weeks | 12 weeks | 14 weeks |
|  |  | (-14 to -1 days) | (0 day) | (28±5 days) | (56±5 days) | (84±5 days) | (98±5 days) |
| Subject consent | | O |  |  |  |  |  |
| Demographic survey | | O |  |  |  |  |  |
| Vital signs | | O | O | O | O | O | O |
| Medical history and disease investigation | | O |  |  |  |  |  |
| Prior/Concomitant Drug Investigation | | O | O | O | O | O | O |
| Laboratory examination^1)^ | | O | O |  |  | O | O |
| Pregnancy test (HCG) | | O | O | O | O | O | O |
| Subject suitability evaluation | | O |  |  |  |  |  |
| Randomisation | |  | O |  |  |  |  |
| Functional test | Kupperman Index | O | O |  |  | O |  |
|  | Menopausal rating scale | O | O |  |  | O |  |
|  | Lipid profile | O | O |  |  | O |  |
| Safety evaluation | Estradiol | O | O |  |  | O |  |
|  | FSH^2)^ | O | O |  |  | O |  |
|  | LH^3)^ | O | O |  |  | O |  |
|  | Mammography | O | O |  |  |  |  |
|  | Thickness of the endometrium, as assessed by ultrasound | O | O |  |  | O |  |
| Intervention provision | |  | O | O | O |  |  |
| Investigation of adverse reactions | |  |  | O | O | O | O |
| Return of intervention tablet and evaluation of compliance | |  |  | O | O | O |  |
| 1) Laboratory examination:   - Complete Blood Count: WBC, RBC, Haemoglobin, Haematocrit, Platelets, Neutrophils, Lymphocytes, Monocytes, Eosinophils, Basophils - Blood chemistry: AST, ALT, r-GTP, ALP, Bilirubin-Total, Total protein, Albumin, TC, TG, HDL-C, LDL-C, BUN, Creatinine, Uric acid, Glucose, CRP, ESR - Urine test: pH, Protein, Glucose, WBC, RBC, Specific gravity   2) Follicle Stimulating Hormone  3) Luteinising hormone | | | | | | | |

**Supplementary Table 3**. Interventional Components

| RCAM tablet | | | Placebo | | |
| --- | --- | --- | --- | --- | --- |
| Raw material name | mixing ratio(%) | Content(mg) | Raw material name | mixing ratio(%) | Content(mg) |
| RCAM  mixed extract | 48.00 | 480.0 |  |  |  |
| Crystalline cellulose  ( MICROCRYSTALLIN) | 46.20 | 462.0 | Crystalline cellulose  ( MICROCRYSTALLIN) | 94.20 | 942.0 |
| Magnesium Stearate | 1.50 | 15.0 | Magnesium Stearate | 1.50 | 15.0 |
| Silicon Dioxide | 1.50 | 15.0 | silicon dioxide | 1.50 | 15.0 |
| Hydroxypropylmethylcellulose | 2.50 | 25.0 | Hydroxypropylmethylcellulose | 2.50 | 25.0 |
| Glycerin fatty acid ester | 0.25 | 2.5 | Glycerin fatty acid ester | 0.25 | 2.5 |
| Cochineal Extract Color | 0.05 | 0.5 | Cochineal Extract Color | 0.05 | 0.5 |
| Total | 100.00 | 1,000.0 | Total | 100.00 | 1,000.0 |

**Supplementary Table 4**. Abbreviations and Glossary of Terms

| ADR | Adverse drug reaction |
| --- | --- |
| AE | Adverse event |
| ALP | Alkaline phosphatase |
| ALT | Alanine aminotransferase |
| ANCOVA | Analysis of covariance |
| AST | Aspartate aminotransferase |
| BUN | Blood urea nitrogen |
| CRP | C-reactive protein |
| ESR | Erythrocyte sedimentation rate |
| E2 | Estradiol 2 |
| FAS | Full analysis set |
| FSH | Follicle stimulating hormone |
| γ-GT | Gamma-glutamyltransferase |
| HCG | Human chorionic gonadotropin |
| HDL-C | High density lipoprotein-Cholesterol |
| ITT | Intention-To-Treat |
| KI | Kupperman index |
| IRB | Institutional Review Board |
| LDL-C | Low density lipoprotein-Cholesterol |
| LH | Luteinising hormone |
| MSR | Menopause Rating Scale |
| PP | Per protocol |
| RBC | Red blood cell |
| RCAM | *Rubus coreanus* Miq. and *Astragalus membranaceus* Bunge |
| TG | Triglyceride |
| UNL | The upper normal limit |
| WBC | White blood cell |
